# Mapping hotspots of malaria transmission from pre-existing hydrology, geology and geomorphology data in the pre-elimination context of Zanzibar, United Republic of Tanzania

**DOI:** 10.1186/s13071-015-0652-5

**Published:** 2015-01-22

**Authors:** Andrew Hardy, Zawadi Mageni, Stefan Dongus, Gerry Killeen, Mark G Macklin, Silas Majambare, Abdullah Ali, Mwinyi Msellem, Abdul-Wahiyd Al-Mafazy, Mark Smith, Chris Thomas

**Affiliations:** Department of Geography and Earth Sciences, Aberystwyth University, Aberystwyth, UK; Environmental Health and Ecological Sciences, Ifakara Health Institute, Ifakara, United Republic of Tanzania; Department of Epidemiology and Public Health, Swiss Tropical and Public Health Institute, Basel, Switzerland; Vector Biology Department, Liverpool School of Tropical Medicine, Liverpool, UK; Zanzibar Malaria Elimination Program, Zanzibar, United Republic of Tanzania; School of Geography, University of Leeds, Leeds, UK; Institute of Biological, Environmental and Rural Sciences, Aberystwyth University, Aberystwyth, UK

**Keywords:** Mosquito breeding habitat, Malaria, Larval source management, Hydrology, Geomorphology, Geology

## Abstract

**Background:**

Larval source management strategies can play an important role in malaria elimination programmes, especially for tackling outdoor biting species and for eliminating parasite and vector populations when they are most vulnerable during the dry season. Effective larval source management requires tools for identifying geographic foci of vector proliferation and malaria transmission where these efforts may be concentrated. Previous studies have relied on surface topographic wetness to indicate hydrological potential for vector breeding sites, but this is unsuitable for karst (limestone) landscapes such as Zanzibar where water flow, especially in the dry season, is subterranean and not controlled by surface topography.

**Methods:**

We examine the relationship between dry and wet season spatial patterns of diagnostic positivity rates of malaria infection amongst patients reporting to health facilities on Unguja, Zanzibar, with the physical geography of the island, including land cover, elevation, slope angle, hydrology, geology and geomorphology in order to identify transmission hot spots using Boosted Regression Trees (BRT) analysis.

**Results:**

The distribution of both wet and dry season malaria infection rates can be predicted using freely available static data, such as elevation and geology. Specifically, high infection rates in the central and southeast regions of the island coincide with outcrops of hard dense limestone which cause locally elevated water tables and the location of dolines (shallow depressions plugged with fine-grained material promoting the persistence of shallow water bodies).

**Conclusions:**

This analysis provides a tractable tool for the identification of malaria hotspots which incorporates subterranean hydrology, which can be used to target larval source management strategies.

## Background

It is becoming more apparent that if we are to achieve the ambitious goal of eliminating malaria, it will be necessary to complement current priority interventions for controlling adult mosquitoes that feed and rest indoors, such as long-lasting insecticide treated nets (LLINs) and indoor residual house spraying (IRS) by targeting vector mosquitoes at aquatic habitats where they are most vulnerable. This can be achieved through larval source management techniques such as environmental management or larviciding [1-8].

Zanzibar has one of the most intensive and successful programs for malaria control and elimination in Africa with LLINs and IRS applied to 90% of all dwellings [9,10]. While dramatically reducing the numbers of anthropophagic vectors, specifically *Anopheles gambiae* s.s. and *An. funestus*, species that are not suited to control by indoor interventions, such as *An. arabiensis,* persist [9]. Larval source management interventions that tackle such behaviourally evasive vectors at their aquatic habitats would be most effective if the habitats to be targeted could be identified beforehand, with simple replicable tools [11].

The distribution and characteristics of aquatic habitats used by malarial mosquitoes for oviposition and larval development are controlled by hydrological and geomorphological processes [5,12-20]. The explicit control of hydrology and geomorphology on the formation of malaria vector habitats means that their distribution will be heterogeneous, enabling the identification of hotspots. This has been exploited in studies that associate malaria transmission rates to proximity to marshland or swamps which tend to be found in areas of topographic convergence, such as valley bottoms, for example in the Western Kenyan Highlands and the Usambara Mountains, Tanzania [21-28].

The main assumption behind the hydrological processes that govern this relationship is that the local water table lies parallel to the ground surface [29] and that subsurface flow pathways follow surface flow directions. However, malaria is also prevalent in limestone dominated (karst) landscapes, including southeast Asia, the south Pacific and coastal east Africa [30-36], where the hydrology is markedly different from previous examples given for the Western Kenyan Highlands and the Usambara Mountains, Tanzania. This is due to the high permeability of limestone rock enabling water to infiltrate into the bedrock to form cave systems, rather than converging in topographic depressions or river channels, resulting in a variable relationship between terrain and water table depth. These environments need to be studied if we are to understand the physical controls behind the formation and persistence of vector habitats in malarial regions where limestone is dominant. This is particularly poignant in east Africa where a large proportion of the population live in cities built in coastal areas dominated by coral limestones.

**Figure 1:**
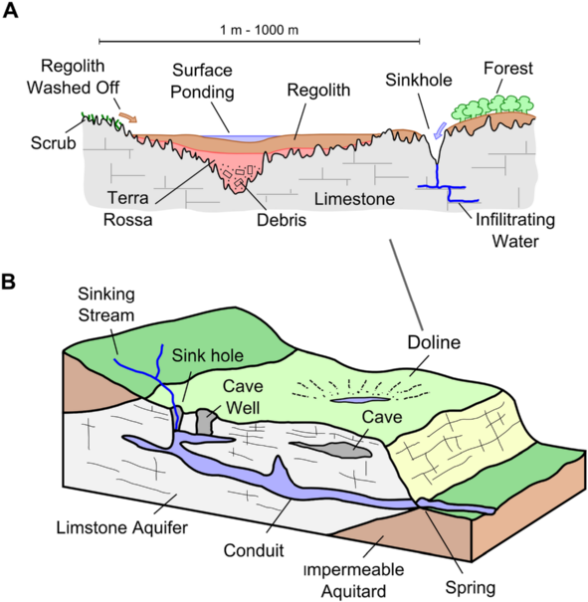
**Key hydrological characteristics on a karst landscape including (A) the deposition of fine-grained material into a doline depression providing a focus for local drainage and (B) the interception of streams in limestones leading to the development of cave systems and ground water aquifers** **[** 39 **]** **.**

In karst landscapes, water reacts with calcium carbonate in limestone, to create a highly corrosive substance (carbonic acid) that exploits natural cracks and crevasses in the rock. Through dissolution widening of fractures, preferential flowpaths and conduits develop in a positive feedback loop eventually leading to the development of sink holes and cave systems (Figure 1A). Rivers and streams intercepting this landscape will be diverted underground (Figure 1A) forming groundwater aquifers, which can re-emerge downstream to feed surface pools of water as natural springs. Corrosion in karst landscapes can lead to the formation of closed bowl-shaped depressions up to 1 km in diameter known as dolines providing foci for local surface drainage [37,38] (Figure 1B). Fine-grained soil and weathered material known as terra rossa, is transported fluvially into dolines [38] and is deposited to form localised areas of low infiltration (Figure 1B) and, in some cases, the development of shallow water bodies [38-40].

Although the hydrology of karst landscapes is well understood, no reported attempt has been made to link this information to aquatic malaria vector habitats in sub-Saharan Africa. In this paper we examine wet and dry season spatial patterns of malaria infection rates reported by health facilities as part of routine surveillance mechanisms on Unguja, Zanzibar which is in the pre-elimination phase and thus making our findings immediately applicable to malaria elimination efforts. Spatial patterns of malaria infection rates are analysed in relation to the physical geography of the island, including its karst geomorphology, hydrology, geology and land cover in order to identify hot spots of persisting malaria transmission.

The aim of this study is to explore the influence of geology, geomorphology and hydrology on patterns of malaria transmission in a karst landscape. The focus of this study is on the limestone dominated island of Unguja, Zanzibar, United Republic of Tanzania, which is in the pre-elimination phase of its Malaria Control Programme.

## Methods

### Study site

**Figure 2:**
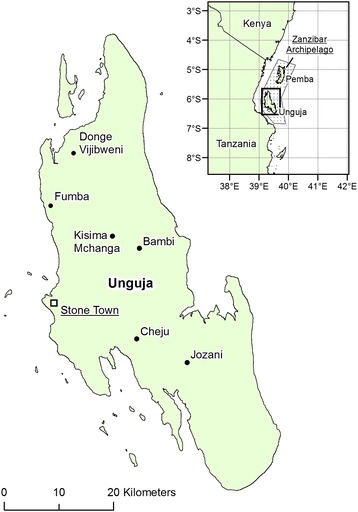
**The location of Unguja, Zanzibar.** Place names include Stone Town, the principal town of the Zanzibar archipelago, and others mentioned in the main text. Source: DIVA-GIS (www.diva-gis.org).

**Figure 3:**
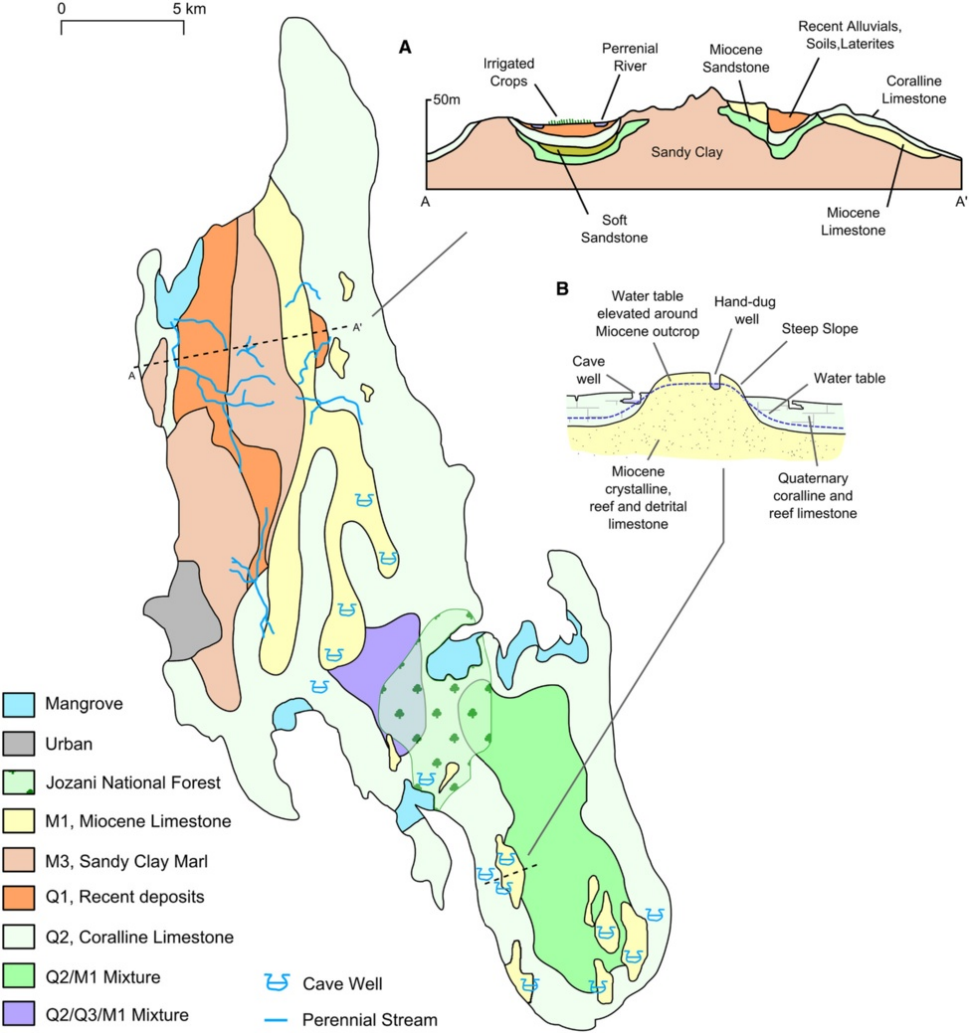
**Geological characteristics of Unguja, Zanzibar, including (A) a cross-section across the northwest of the island, and (B) a diagram demonstrating the effect of dense Miocene limestone outcrops on the water table** **[** 45 **]** **.** See Table 1 for a description of these rock types.

Unguja is the largest island (1600 km^2^) of the Zanzibar Archipelago, located 40 km off the east coast of Tanzania (Figure 2). The island is underlain by Miocene sandy clay marl (Figure 3A). Alluvial deposits and laterites are found on the northwest part of the island up to 130 m above sea-level (Figure 3A). This area supports a small number of perennial rivers and numerous seasonally active streams (Figure 3A), which tend to divert into the ground once they intercept the porous Quaternary coralline limestone reef terraces (Figure 1A) that dominate the rest of the island, particularly in the east and southeast [41] (Figure 3). Over these limestones the landscape is typical of karst environments with the development of sink holes, caves, and doline features. A description of these features and other geographical terms, including rock types, can be found in Table 1.

**Table 1:** **List of geographical terms, including rock type, with a short description and characteristics**

**Name**	**Short description**	**Characteristics**
M1	Crystalline, reef and detrital limestone	Hard and dense crystalline Miocene limestone consisting of broken limestone, crushed coal, shell fragments and bands of flint. Sandy and gritty, formed as discontinuous reef, cavernous in places. Supplies water to lakes at Bambi and many springs and well, including cave wells.
M3	Marls, sandy clays and clayey sands	Forms the main base rock of Unguja. Bluish grey to bluish green in colour comprising of dense, roughly sorted Miocene chalky rocks with veins of gravel which weather to a red, yellow, or brown colour.
Q1	Soils, laterites, alluvial and colluvial deposits	Mixture of red, brown and black Quaternary soils rich in iron oxide typical of tropical environments. This fine grained soil maintains a water table forming an underground aquifer which provides a source of water for hillside springs.
Q2	Coralline and reef limestone	White, cream or yellow-brown Quaternary limestone which tends to be grey along rocky and jagged outcrops. Notably free from iron staining. Common across Unguja, except the north-eastern region, forming the island’s main underground aquifer. Frequently cavernous forming many cave wells in conjunction with M1.
Q3	Marine and fluvial sands and sandstone	Sands mixed with shell fragments, fish bones and sharks’ teeth which are lightly cemented forming grey, coarse Quaternary sandstone. Provides water for pumped wells at Kisima Mchanga and Cheju.
Doline	Bowl-shaped depression	Bowl-shaped closed depressions (1–1,000 m in diameter) formed by the dissolution of limestone rocks by corrosive groundwater (carbonic acid from the reaction of water with calcium carbonate which is abundant in limestone rocks). Fine-grained soils often drain into these features.
Infiltration	The rate at which a soil or rock is able to absorb water	Low infiltrating soils on Unguja are relatively fine grained, well-weathered soils typical of the Q1 geology type. Rainwater and irrigation will absorb relatively slowly into the soil helping to keep soils saturated and retain water at the surface. Conversely, the Q2 rock type is characterised by high infiltration due to cracks and crevasses.
Regolith	Fine-grained weathered material	Loose, fine-grained material formed by weathering of rocks.
Terra rossa	Red clay soil	Red clay soil produced by the weathering of limestone.
Perennial stream	A river channel that runs continuously throughout the year	

See Figure 3 for a map of geology types.

Unguja receives between 1000 and 2250 mm of rainfall per year. Rainfall is strongly seasonal, typically with dry and hot weather during January and February, heavy rains from March to May, a dry season during June to September and light rains during October to December [42]. The vegetation of the island comprises secondary forest, mangrove swamps, and degraded fallow bush. Agriculture is mainly dominated by root crops, vegetables and rain-fed rice plantations [43]. In some areas, low infiltrating soils and the availability of groundwater supply supports irrigated rice and sugar cane plantations. Intense deforestation in advance of agricultural development has left little original vegetation with the exception of the Jozani Forest National Park [42] which is located within a shallow basin where the water table is relatively high.

### Malaria infection rates

Data on malaria cases for the 2011 wet season (April-June) and dry season (July-September) are based on weekly summary reports of numbers of suspected, tested and treated malaria cases amongst patients from 144 health facilities (49 located on Unguja) located across the Zanzibar islands [10]. Transmission data since 2011 was not available. Data prior to this year was not considered in the analysis because patterns of malaria transmission will be strongly affected by a campaign distributing treated bed nets and indoor residual spraying. At the health facilities, patients presenting signs and symptoms of malaria, including history of fever, were tested for malaria using either quality assured microscopic examination of Giemsa-stained thick blood films or a Rapid Diagnostic Test (Paracheck® or SD Bioline®) [10]. To enable binomial analysis of the data, malaria positivity rates (number of patients tested positive for malaria/the number of patients tested) were classified as hotspot or non-hotspot based on whether they are above or below the mean rate, following the example of Bousema *et al*. [44]. Information regarding the condition of the facility is also recorded and used as a control variable in subsequent analyses. Facility condition was categorised as very bad, bad, good and very good by the Zanzibar Ministry of Health according to the infrastructure (electricity, safe water supply, telephone), services and care offered (basic equipment, laboratory capacity, infection control), and treatments administered (HIV/AIDS, mental health, diabetes, tuberculosis) at the facility.

### Physical geography data

**Figure 4:**
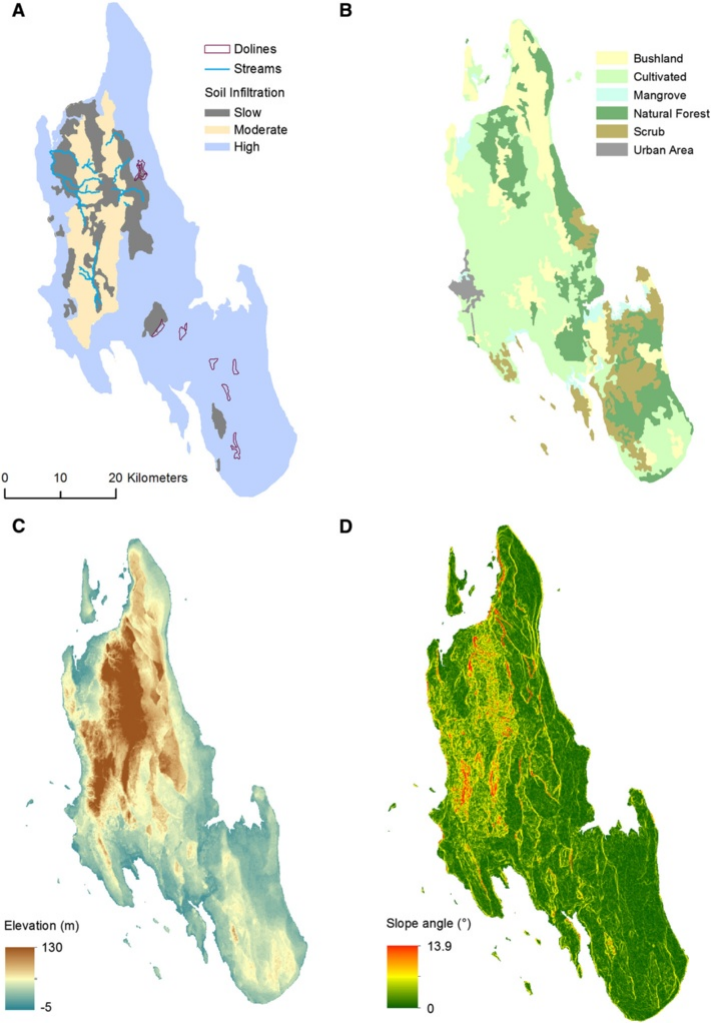
**Variables describing the physical geography of Unguja, Zanzibar including (A) soil infiltration (including the location of streams and dolines)** **[** 41 **,** 46 **]** **, (B) land cover type (Zanzibar Water Authority), (C) SRTM elevation** **[** 47 **]** **and (D) slope angle.**

Datasets describing the physical geography of Unguja were obtained following a study into the water resources of the island [43], including geology (Figure 3) [45], the location of streams and dolines [41] (Figure 4A), and soil infiltration rate [46] (Figure 4A). Additional geological and water resource information, including land cover type (Figure 4B), was obtained from the Zanzibar Water Authority. Elevation data from the Shuttle Radar Topography Mission (SRTM) was downloaded from United States Geological Survey online data archive Earth Explorer [47] (Figure 4C). This was used to calculate slope angle (Figure 4D) using ArcMap 10.1 [48]. To facilitate spatial analyses all data were converted to raster datasets with a spatial resolution of 90 m, the rivers and dolines data were summarised using the Distance to Feature tool in ArcMap. Streams and rivers can provide aquatic habitats for malaria vector larval development [14,15,17] meaning that proximity of households to river features may increase the risk of malaria transmission. There have been no documented attempts to link the presence of doline landforms to malaria incidence, though their characteristics, as shallow inwardly draining basins [37,38], make them conducive to supporting vector larval development. The distance to feature rasters for both dolines and rivers will test whether proximity to these features, and their potential for providing malaria vector habitats, affects the intensity of malaria transmission. Values for each of the physical geography raster datasets were extracted at the location of each health facility using the Extract Multi Values to Points tool in ArcMap using bilinear interpolation of the raster cells adjacent to the central cell at the location of each facility. The resulting dataset is summarised in Table 2.

**Table 2:** **Variables used for modelling hotspots of malaria infection using datasets describing the physical geography of Unguja, Zanzibar**

**Variable**	**Description**	**Mean (range)**
*Dependent variables*		
Apr-Jun 2011	Binary malaria infection hotspot/non-hotspot (n = 49) for the wet season	
Jul-Sep 2011	Binary malaria infection hotspot/non-hotspot (n = 49) for the dry season	
*Independent variables*		
Geology	Geology type:	
	M1: hard, dense crystalline Miocene limestone	
	M3: dense Miocene chalky rock	
	Q1: Quaternary tropical laterites	
	Q2: Quaternary limestone	
	Q2M1: mixture of Q2 and M1	
	Q2Q3M1: mixture of Q2, Q3 (lightly cemented Quaternary sandstone) and M1	
Distance to streams	Distance to perennial streams (m)	12251 (154–36096)
Distance to dolines	Distance to dolines (m)	9746 (0–26685)
Infiltration	Soil infiltration rate: 1 = low, 2 = medium, 3 = high	
Landcover	Land cover type: B = bushland, C = cultivated, F = natural forest, M = mangrove, S = scrub, U = urban	
Elevation	Elevation (m) above sea level	28.5 (8–99)
Slope	Slope angle (°)	1.5 (0.3-4.5)
*Control*		
Condition	Condition of health facility: 1 = Very Bad, 2 = Bad, 3 = Fair, 4 = Good, 5 = New	

Some of the variables listed in Table 2 do not necessarily have a direct biological explanation for the presence or absence of malaria transmission hotspots. For instance, the maximum values for the variables distance to dolines and distance to perennial streams (36 km and 26.7 km respectively) far exceed the maximum flight distance of an adult anopheline mosquito [49]. Rather, these distances represent the highest single values for any distance separating these landscape features from the nearest health facility. The statistical tests employed in this study rely on all the data encompassing the full range of distances between pairs of geographic locations where these dependent and independent variables were observed. We cannot therefore infer that any single geographic feature has a statistically significant impact on malaria risk in its own right.

Despite this, the human malaria infection indicator used this study was diagnostic positivity as recorded at health facilities where patients self-reported at their own discretion, actively travelling to their own preferred choices among these units of observation over a range of distances. The distribution of observed hot spots is therefore not equivalent to the distribution of transmission exposure determined by mosquito proliferation and dispersal alone, but also to human mobility. As such, the landscape features examined in this study could affect malaria infection risk observed at health facilities beyond the normal flight range of mosquitoes.

### Data analysis

The relationship between the malaria positivity rates and the physical geography descriptors was explored using boosted regression tree (BRT) analysis fitted in R [50] using the ‘dismo’ package [51]. This type of modelling generates a robust estimate of response variables by combining a large number of simple models, or regression trees [52-54] which has advantages over Generalised Additive Models or multiple linear regression models that relate the response variable to a number of predicting variables via a single model with well documented drawbacks, including bias in parameter estimation and inconsistencies in model selection [53,55-57].

The binary wet and dry season malaria infection hotspot data were linked to the physical geography variables using a binomial BRT model fitted with a Bernoulli distribution. The BRT models were fitted using a tree complexity of 5 and a learning rate of 0.002 which fitted a minimum recommended number of trees (>1000) while providing optimal training and cross-validation AUC scores [53,56]. A bag fraction of 0.5 was used following Elith *et al*. [56].

The BRT procedure returns a list of the predicting variables ranked by their relative contribution (%) to the model which is used to evaluate the influence of each predicting variable on the model. The relative contribution represents the number of times the variable was selected for splitting, weighed by the improvement to the model as a result of each split averaged over all trees [56,58]. Model predictive performance is evaluated using the area under the receiver operating curve (AUC) reported for training of the model and subsequent cross-validation [53,56].

The BRT modelling procedure is used in this instance to form a correlative model between hotspots of malaria infection and metrics summarising the physical geography of the island. As such, we may only infer but not directly imply causal relationships between these metrics and the abundance of malaria vectors, and of course any such statistical associations may be spurious, particularly those with modest levels of significance. Nevertheless, this robust statistical approach can be used to illustrate inherent spatial heterogeneity in the distribution of malaria transmission through a process-based understanding of the physical hydrological and biological mechanisms taking place in the landscape and how this can be exploited in future integrated malaria control initiatives.

### Ethical consideration

The malaria infection data used in this study was collected as part of a routine surveillance initiative by the Zanzibar Malaria Control Program and was not collected for research purposes and is fully anonymised with no personal identifiers. These records are centred on health facilities so residence is also anonymised.

## Results

**Figure 5:**
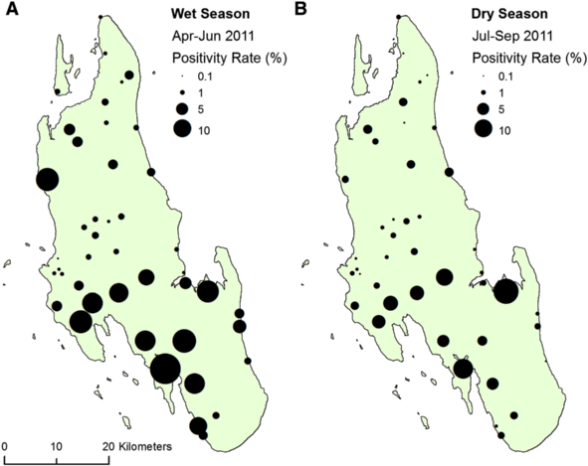
**Maps of malaria positivity rates recorded at health facilities across Unguja, Zanzibar for the 2011 wet (A) and dry (B) seasons displayed as proportional symbols.** Data from the [10].

The total number of patients tested for malaria infection in the wet season (April-June) of 2011 was 40,173 with 3.2% being diagnosed with malaria. This rate decreased in the dry season (July-September) of 2011 with 1.5% of 25,858 patients being tested positive for malaria. The mean malaria infection rate across the health facilities was 4.5% for the wet season and 2.6% for the dry season. Malaria positivity rates varied in health facilities across Unguja (Figure 5) with a tendency for higher rates to be found in the south of the island and lower rates found in the north. The spatial distribution of malaria infection rates was similar in both the wet and dry season with a Pearson’s correlation, corrected for spatial autocorrelation [59,60], of 0.71 (P < 0.001).

The BRT model for wet season hotspots of malaria transmission was fitted with 1600 trees and the dry season model was fitted with 1350 trees. AUC scores were significantly better than random with a cross-validation AUC of 0.89 and 0.8 for the wet and dry season models respectively (Table 3) indicating that hotspots of malaria infection were successfully modelled using the physical geography variables outlined in Table 2.

**Table 3:** **Boosted Regression Tree modelling results for predicting hotspots of malaria infection for the 2011 dry and wet seasons using variables describing the physical geography of Unguja, Zanzibar**

**Wet season**	**Number of trees fitted = 1600**	
	**AUC**	**Std. Error**
Calibration	0.9	
Cross-validation	0.89	0.05
**Dry season**	**Number of trees fitted = 1350**	
	AUC	Std. Error
Calibration	0.86	
Cross-validation	0.8	0.075

The relative influence of each variable on the wet and dry season BRT models were similar with distance to dolines and slope angle having a high influence and soil infiltration rate having little or no influence (Table 4). Malaria transmission hotspots were negatively associated with distance to dolines which is supported by Pearson correlation coefficients of −0.33 and −0.34 (P value < 0.05) for malaria positivity rates in the dry and wet season respectively. Although, malaria transmission hotspots were not significantly univariately correlated with distance to streams, a positive relationship (more malaria further from streams) had a high influence in the BRT model. A spatially corrected Pearson’s correlation indicated that the metrics distance to dolines and distance to streams were not significantly autocorrelated with a correlation coefficient of −0.42 (P > 0.05).

**Table 4:** **Contributions of predictor variables to the boosted regression tree models predicting physical geography variables to wet season and dry season hotspots of malaria infection on Unguja, Zanzibar**

**Variable description**	**Relative influence (%)**	
	**Wet season**	**Dry season**
Slope angle (°)	28.7	25.6
Distance to doline (m)	25.6	35.1
Distance to stream (m)	18.2	8.7
Landcover type	13	17.4
Geology type	10.1	8.3
Elevation (m)	3.9	4.7
Soil infiltration rate	0.5	0.1
Condition of health facility	0	0

**Figure 6:**
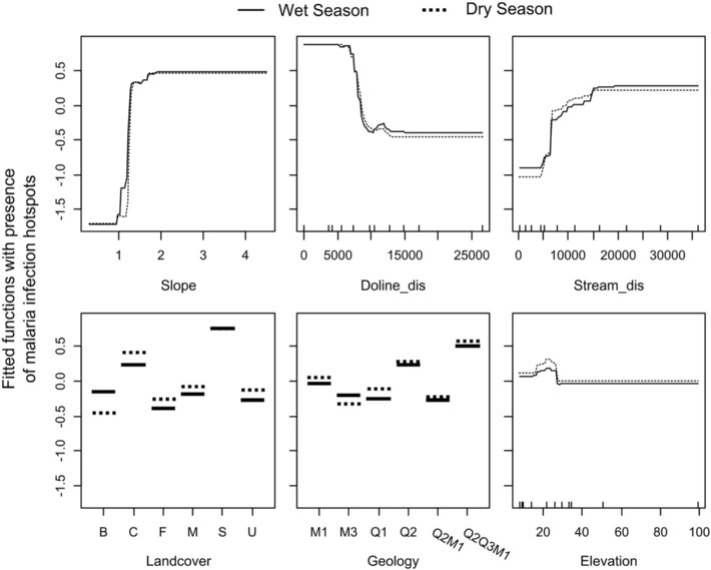
**Fitted function plots for the independent landscape variables in the model for predicting 2011 wet and dry season malaria infection hotspots on Unguja, Zanzibar.** B = bushland, C = cultivated, F = natural forest, M = mangrove, S = scrub, U = urban. See Table 2 for a description of other variables and units. The variables soil infiltration rate and health facility condition are not included due their negligible influence on the models (see Table 4).

The fitted functions for the predicting variables were similar for both wet and dry season models (Figure 6) although some small differences existed for the landcover variable. Specifically, areas of bushland were less associated with malaria infection hotspots in the dry season compared to the wet season. Conversely, cultivated land was more associated to malaria infection hotspots during the dry season, relative to the wet season. The scrubland landcover type had the greatest association with hotspots of malaria transmission. Geology had a similar level of influence in both the wet and dry season models. Of the different geological types, coralline and reef limestone (Q2) and the mixture of crystalline, reef and detrital limestone, with marine and fluvial sands and sandstone (Q2Q3M1) was shown to have the clearest relationship with hotspots of malaria transmission. The condition of the health facility had no influence on either the wet or dry season models meaning that subsequent inferences are independent from the state of facilities at which malaria incidence is reported.

**Figure 7:**
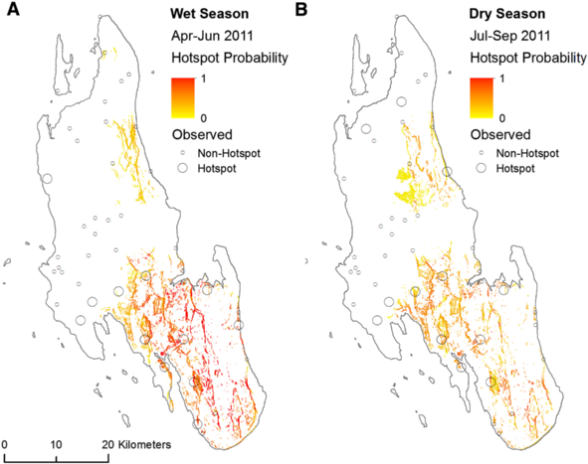
**Observed hotspots of malaria infection and probability of malaria infection hotspots predicted from a BRT model using variables summarising the physical geography of Unguja, Zanzibar for (A) the wet season and (B) the dry season.** White areas are where probability of malaria infection hotspot was predicted to be −1 to 0.

Predicted maps of malaria hotspot probability are shown in Figure 7. Predictions for both the wet and dry seasons indicate similar patterns, with an increase in predicted hotspots in the south of the island and the northeast also demonstrating potential for hotspot occurrence. Primarily, hotspots are concentrated in areas with steep slopes close to dolines. The model follows observed hotspots for most locations across the island, although some apparent false negatives occur. For instance, hotspots occur at Fumba on northwest coast and Donge Vijibweni in the far north of the island (see Figure 2 for locations) but are not predicted by the model. Some false positives also occur, particularly in areas with steep coastal cliffs, such as Pwani, where slope angle has a high influence on both the wet and dry season BRT models.

## Discussion

Relatively high malaria infection rates were reported in health facilities towards the south and central part of the island with relatively low rates in the north (Figure 5). The south and central part of the island is dominated by limestones with high infiltration rates where surface water tends to be diverted underground, illustrated by the influence of coralline and reef limestone (Q2) in both the wet and dry season BRT models (Figure 6) which forms the island’s main aquifer complex [45]. The dominance of groundwater in this area provides a temporally stable supply of water to malaria vector habitats independently from rainfall. Additionally, this area includes outcrops of dense limestone which maintain relatively high water tables feeding surface water bodies including cave wells and hand dug wells [45,61] (Figure 3B). These outcrops are represented by the rock complex Q2Q3M1 (a mixture of crystalline, reef and detrital limestone, with marine and fluvial sands and sandstone) which was shown to influence the BRT models (Figure 6). These outcrops are characterised by steep slopes, resulting in the variable slope angle being one of the most influential variables for predicting dry and wet season malaria infection hotspots (Table 4).

Distance to dolines was also an important variable for predicting hotspots of malaria infection (Table 4) as high concentrations of these landform features occur in the central and southern part of the island, providing localised depressions for the formation of surface water bodies [39]. The locations of these landforms often coincide with areas of scrub vegetation, which was shown to be positively associated with hotspots of malaria infection (Figure 6). This landcover type typically occurs at the fringes of forested areas, such as the groundwater-fed Jozani National Forest [42,43,45], and is characterised by a fine-grained red-brown sandy top soil [42] that often occupies crevices in the limestone rock [46] providing a focus for local drainage and the development of shallow pools [38-40].

Elevation had a marginal influence on both the wet and dry season BRT models of malaria infection hotspots (Table 4). This reflects the influence of subterranean processes governing the movement of water on Unguja through the karst landscape via an aquifer network or the concentration of water bodies in localised areas of low infiltration, such as doline features infilled with fine grained material. This is in contrast to other, non-karst landscapes, such as the Western Kenyan Highlands and the Usambara Mountains, Tanzania, are dominated by the overland, or near-surface movement of water, resulting in an association between elevation and/or terrain and malaria transmission [21-28].

Distance to streams was a relatively important predicting variable (Table 4) with hotspots of transmission being associated with large distances from perennial streams and rivers (Figure 6). The positive relationship suggests that while streams on Unguja may support malaria vector larval development, their suitability is low and is probably outweighed by their ability to provide habitats in the surrounding floodplains due to flooding. This is likely to be particularly important in the wet season when increased flows not only increase the suitability of the floodplains, but also make habitats located within river channels less suitable for anopheline larvae due to habitat flushing [62,63] and an intolerance of fast flowing water [2,63,64]. Additionally, pollution, due to the absence of sewerage infrastructure [65], may also negatively influence the ability of rivers to support vector larval development [66]. Additional work focussed on this habitat type is needed to establish their ability to support malaria vector larvae particularly where channels of similar dimensions (approximately 10 m in width and 5 m deep) in the Kilombero Valley southern Tanzania have been found to support vector larval development within chains of pools that form in the river bed once the river ceases to flow in the dry season [12]. The contrast between these two distinct ecosystems merits consideration: In contrast to Unguja, in the Kilombero Valley floodplain, extensive aquatic habitats are formed during the wet season, when the floodplains of these channels are inundated with water during peak river flows, while in the dry season, river channels provide suitable habitats in chains of pools that form in the river bed once the river ceases to flow [12]. Specifically, this function (the formation of habitats in river channels during the dry season) has the potential to support dry season transmission, making it a crucial process for maintaining malaria endemicity throughout the year. As such, the potential for streams and rivers on Unguja to provide productive malaria vector habitats cannot be dismissed and requires further detailed investigation.

We found that the spatial pattern of malaria infection remained relatively static during the 2011 wet and dry seasons. Given the pronounced seasonality to rainfall patterns in the Zanzibar islands this suggests that aquatic malaria vector habitats are chiefly fed by groundwater sources which persist throughout the year. The influence of groundwater processes dominating the formation and persistence of water bodies means that geology and hydrological processes need to be understood if the dynamics of aquatic malaria vector habitats are to be mapped and targeted for larval source interventions.

Malaria infection rates on Unguja demonstrated spatial heterogeneity which can reduce the effectiveness of control strategies where resources are wasted on areas with low or negligible rates of malaria [67]. It is therefore crucial to identify geographic foci of disease transmission for targeting interventions [44,68]. Bejon *et al*. [67] noted the uncertainty surrounding the temporal stability of disease hotspots at a vector dispersal level (0.5-1 km) in Kenya. For Unguja, patterns of malaria infection were similar in both the 2011 wet and dry seasons, increasing the potential for efficient targeting of interventions. Targeting malarial habitats in this way may help to eliminate vector species, such as *An. arabiensis*, that are not vulnerable to conventional indoor-based interventions [9] and help to eliminate malaria on Unguja.

Relatively low malaria infection rates were found in the central northern part of the island in 2011. The availability of surface water in this region of the island, owing to the dominance of low infiltrating sandy clay soils and recent alluvial deposits (Q1, Q3 and M3) [45,46], is exploited by widespread cultivation of crops including sugar cane and rice. Saturated rice fields often support high vector abundances, particularly for species such as *An. arabiensis* which are the dominant vector on the island [9]. Despite this, wards within this region demonstrated low infection rates in both the wet and dry seasons (Figure 5). Reasons behind the reduction in malaria positivity rate in this region of island remain unclear. Some authors have purported a ‘paddies paradox’ whereby reduced malaria transmission has been associated with irrigated crop production in areas with stable transmission due to a focus of indoor-based vector control interventions [69,70]. However, we do not believe this is the case on Unguja where there are no distinct geographical differences in the use of LLINs and IRS [9,10]. From our study we hypothesise that these habitats are in fact relatively unproductive for vector mosquitoes, but this needs to be tested by entomological survey. Although irrigated areas showed no association with malaria, hotspots were more likely to be found in areas of cultivated land during the dry season. These areas are likely to be occupied at night during certain points of the year to protect seedlings from birds, or farmers working late for ploughing or harvesting, increasing their risk of exposure to malarial mosquitoes [71]. The apparent seasonal independence of the spatial distribution of malaria transmission on Unguja is testament to the notion that dry season transmission is more important for sustaining endemicity than wet season transmission and should, therefore, provide encouraging opportunities for focussing seasonal malaria control activities at exactly the time of year when they are easiest to implement [72,73].

**Figure 8:**
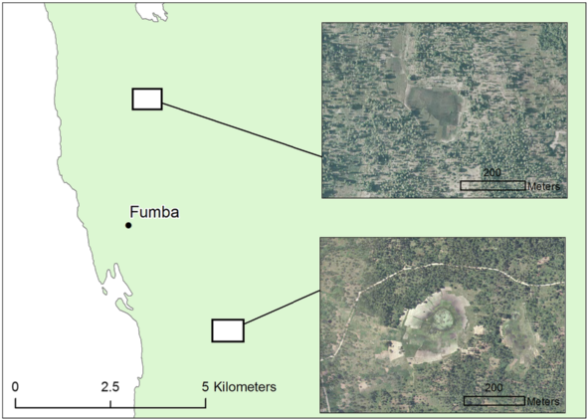
**Malaria transmission hotspot located at Fumba (see Figure** **2** **for location within Unguja).** Sub-panels show 50 cm aerial imagery of water bodies located within doline landforms close to the health facility.

**Figure 9:**
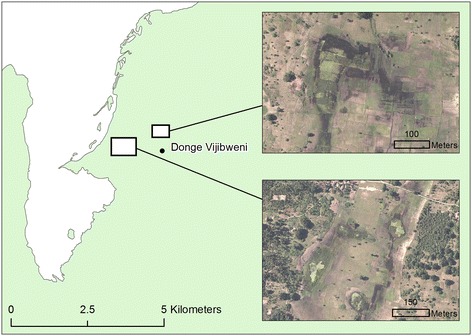
**Malaria transmission hotspot located at Donge Vijibweni (see Figure** **2** **for location within Unguja).** Sub-panels show 50 cm aerial imagery of water bodies located within doline landforms close to the health facility.

Despite the general trend of relatively low malaria transmission rates in the northern part of Unguja, two hotspots of malaria infection occur in this region: at Fumba on the northwest coast (Figure 8) and Donge Vijibweni in the far north of the island (Figure 9). Both health facilities are located close to relatively small doline (400 m wide) features which have not been identified in the environmental layers obtained from the Zanzibar Water Authority. In addition, both sites are located in close proximity to streams, the latter having a negative association with malaria infection rates for most hotspots on the island. Such areas could be mapped using high spatial resolution (<2 m) aerial/satellite imagery, or through using remote sensing systems such as L-band Radar [74], with contextual information to differentiate from anthropogenically induced wetted areas, such as irrigated rice paddies. Interestingly, both these locations are known hotspots of malaria transmission highlighted at the onset of the Zanzibar Malaria Control Programme’s Malaria Epidemic Early Detection System [75] and remain despite extensive distribution of indoor-based interventions.

This study identified two key processes that are enabling the development and persistence of malaria vector habitats, particularly in the south and central part of the island where malaria infection rates remain high. Firstly, outcrops of dense crystalline, reef and detrital limestone (M1), intercepting the main groundwater aquifer complex, maintain relatively high water tables, increasing the potential for surface water bodies to persist [43,45]. Secondly, dolines, which are naturally occurring depressions in limestone dominated karst landscapes, can become plugged with fine-grained material which is washed in from the surrounding area leading to pockets of low infiltrating soils providing a focus for local drainage and the development of shallow pools [38-40] that are likely to be suitable for vector oviposition and larval development [28]. Using available static geological maps we were able to identify areas where these two processes exist representing a significant tool for informing larval source intervention strategies.

The accumulation of fine-grained material and subsequent development of shallow water bodies is also prominent at the fringes of the Jozani National Forest [42,43,45]. Deforestation, which has been prevalent on the island [42], is likely to increase the availability of fine-grained material [76], potentially increasing the coverage of low infiltrating soils with a propensity to support surface water bodies and potential habitats. This process been linked to a rise in historical malaria cases in ancient Rome [77,78], as well as more recent examples [79,80] and should provide a lesson for present and future land managers in malarial regions [79,81].

### Study limitations

The malaria infection rates used in this study are based on summary reports from health facilities. This makes the assumption that the landscape, and therefore physical geography variables, is the same where the patient lives. Given the heterogeneous nature of the landscape across Unguja this is likely to introduce uncertainty. Using information regarding the specific location of patient dwellings would provide a more precise indication of the role of physical geography on the risk of being infected with malaria. Furthermore, this may help to identify specific breeding habitats prolific in malaria vector production which can be used to target larval source management interventions. Data from a greater number of sites will also improve the spread across the different physical geography components across the island. For instance, few health facilities were located close to mangroves and therefore this landcover type is not identified in our study as being as related to malaria infection. In this specific case, the analysis may not take into account malaria transmission due to salt-tolerant vectors found in mangrove forests, such *An. merus* [82]. This is not assumed to have a significant impact on the study findings because *An. merus* contributes a relatively small proportion (5%) of adult mosquitos sampled on Unguja in 2010 [9]. Additionally, the data used in this study does not take into account patients that contracted malaria away from their dwelling. Information regarding the travel history of the patient should be gathered to take this aspect into account, although research has indicated a relatively low risk of importing malaria from mainland Tanzania [83].

Our study indicates that proximity to streams is negatively correlated with hotspots of malaria infection. Despite this correlation we do not suggest that habitats located in river channels do not represent important breeding sites. In particular, whereas steep sides, strong flows, an abundance of predators and other contributing factors have the potential to make streams less likely to support malaria vector habitats during the wet season [2,63,64], streams during periods of low flow have been shown to support vector larval habitats, particularly in during the dry season in ephemeral channels when the river stops flowing, forming small, shallow water bodies in the river bed [12]. Additionally, a number of sources indicate the importance of shallow pools of water forming along the periphery of rivers [5,13-18,84], particularly following periods of overbank flooding, with the potential to support vector larval development. To this end, the function of streams on Unguja on supporting malaria vector populations needs to be explored further.

## Conclusions

This study has demonstrated the effects of hydrology and geology on the distribution of malaria transmission hotspots. It shows that patterns of malaria infection gathered using a routine programmatic surveillance platform demonstrate disease heterogeneity on Unguja, Zanzibar, United Republic of Tanzania. Furthermore, spatial patterns of malaria infection can be predicted using static data, such as geology and slope angle, which underpin the hydrology of the island, helping to develop a framework for targeting malaria vector habitats for intervention strategies. These methods can be applied at the national scale where similar surveillance programmes exist, such as Zambia [85] and the Solomon Islands [86], to provide fine-scale information for malaria elimination campaigns. All of the data used in this work was freely available, providing an example of the great potential for replicating the methods outlined in this paper for similar landscapes burdened by malaria.
